# Simulated Performance of Laser-Machined Metamaterial Anti-reflection Coatings

**DOI:** 10.1007/s10909-022-02751-7

**Published:** 2022-06-15

**Authors:** N. Farias, S. Beckman, A. T. Lee, A. Suzuki

**Affiliations:** 1 Department of Physics, University of California, Berkeley, Berkeley, CA 94720 USA; 2 Lawrence Berkeley National Lab, Berkeley, CA 94720 USA

**Keywords:** Cosmic microwave background, Anti-reflection coating, Metamaterial, Laser etching

## Abstract

Lenslet-coupled antenna arrays have been used in CMB experiments and are the baseline technology for the next-generation satellite missions such as LiteBIRD and PICO. Lenslets are small hemispherical lenses mounted on the focal plane that couple light to the detectors and are typically made of silicon or alumina due to their high focusing power and low absorption loss. To minimize reflection at the vacuum-dielectric interface, lenslets require anti-reflection (AR) coatings. Metamaterials have been used in large microwave optical components because they avoid any mismatch on the thermal expansion between the lens and its coating, but so far they have only been machined on surfaces of comparatively large radius of curvature. As a first step to understand the feasibility of machining metamaterial AR layers in lenslets through laser-etching for the LiteBIRD mission, a model in ANSYS HFSS was developed. The goal of the simulation was to optimize transmission in three frequency bands while meeting assumed laser machinability constraints and optical requirements. Simulation results from flat silicon show that an AR metamaterial coating made under the assumed conditions is feasible, and the baseline parameters for further curved-surface studies are provided.

## Introduction

LiteBIRD is a satellite mission that will map the B-mode polarization of the cosmic microwave background (CMB) with the main objective of carrying out a definite search for the signal from cosmic inflation [1]. The satellite will contain three telescopes: the low-frequency telescope (LFT), the mid-frequency telescope (MFT), and the high-frequency telescope (HFT), which combined will detect radiation in bands ranging from 34 to 488 GHz.

**Fig. 1:**
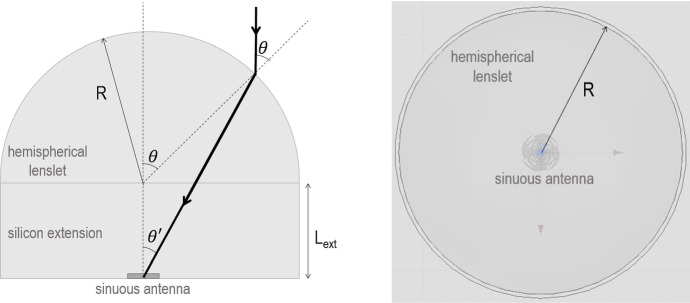
Illustration of the approximate ray path through the lenslet (left) and top view (right) of a LiteBIRD lenslet coupled to a sinuous antenna

The LFT and MFT focal plane modules consist of lenslet coupled sinuous antenna transition-edge sensor (TES) bolometer arrays, as pictured in Fig. 1 [2]. Lenslets are hemispherical lenses that received their name due to their small size ${\mathscr {O}}$(few centimeters) compared to other optical components in modern CMB experiments. In LiteBIRD, lenslets will be made of silicon due to its high refractive index ($n_{\rm Si} \approx 3.4$) and low loss ($tan\delta \approx 10^{-5}$) in the microwave spectrum at cryogenic temperatures [3]. Silicon’s high refractive index is desired due to its high forward gain, but it also results in significant photon reflection at the vacuum interface of roughly 30%, which decreases the overall instrument sensitivity.

**Table 1:** LiteBIRD lenslets frequency bands

Telescope	Pixel name	Pixel size [mm]	Frequency range [GHz]	Frequency band abbreviation
LFT	LF-1 and LF-2	32	34–99	LF-12
LFT	LF-3 and LF-4	16	60–162	LF-34
MFT	MF-1 and MF-2	12	77–224	MF-12

To mitigate the optical power loss, LiteBIRD lenslets will be coated with anti-reflection (AR) layers. To achieve high bandwidths, multi-layer AR coatings can be implemented, and optimization algorithms can be used to find the refractive indices and thickness of each layer to maximize transmission in the desired spectrum (see Sect. 2). In the past, lenslets have been coated with dielectrics that have close to optimal indices of refraction such as epoxy [4, 5] and dielectric PTFE [6]. However, coating optical components with multiple different materials can be challenging due to differences in coefficients of thermal expansion (CTEs), which can cause delamination of the AR layer during a thermal cycle.

To avoid separation between the optical components and their AR coatings at low temperatures, CMB experiments have used sub-wavelength features (henceforth metamaterials) as effective dielectric layers at the lens surface. Metamaterials have been fabricated using dicing blades [7, 8] and laser etching [9–12], but to the authors’ knowledge, they have only been machined in optical components with radii of curvature that are orders of magnitude larger than that of the lenslets. By taking advantage of the flexibility in machining different shapes with laser etching, and using a 6-axis table to orient objects, the authors expect to be able to etch AR structures normal to the surface of the highly curved silicon lenslets.

The goal of this study was to investigate at a first level whether laser-etched metamaterials AR layers can be used to increase transmission power through the LiteBIRD lenslets and other experiments such as PICO [13] that will also use lenslet-coupled sinuous antenna pixels. We established a desired minimum transmission of 95% across each LiteBIRD lenslet band to match benchmark experiments [8, 10] of similarly large fractional bandwidth. The bands were defined based on pixel (and thus lenslet) sizes, as reported on Table 1. As the initial step of this investigation, the metamaterial performance was simulated in flat silicon. The transmission through two simple different geometries, conical and cylindrical, was optimized under some assumed machinability constraints.

## Methods

**Fig. 2:**
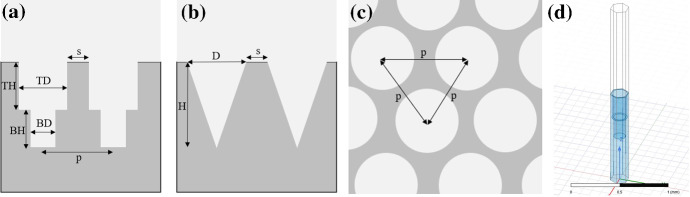
Sketch of side view of **a** 2-layer cylindrical coating, and **b** conical coating, **c** top view showing hexagonal pattern of structures, **d** HFSS model with Floquet ports simulating the performance of two layers of cylindrical holes (Color figure online)

The metamaterial transmission simulations were done using ANSYS high frequency simulation software (HFSS). The setup included a flat silicon substrate of dielectric constant 11.7 and loss tangent $5\times 10^{-5}$ and a surrounding vacuum. The metamaterials were modeled in two hole geometries: conical and cylindrical, as depicted in Fig. 2. The geometries were chosen due to their simplicity, which is an important factor when machining highly curved surfaces. Simulating an entire lenslet with thousands of metamaterial features would take a computational effort beyond what was available to this study. Instead, to account for the effect of the lenslet curvature on the transmittance, we used HFSS’ feature of Floquet ports to simulate an infinite array of repeated cells with periodic boundaries and rays incoming at various angles of incidence.

The modeled geometries had to meet some initial machinability limitations and optical constraints criteria. To prevent diffraction by the metamaterial array, the pitch (distance between structures) should satisfy [14]:

1 $$\begin{aligned} p < \frac{\lambda }{n_{\rm Si} + n_{vacuum} {\rm sin}(\theta _i)} \end{aligned}$$

where $\lambda$ is the smallest wavelength in each frequency band, and $\theta _i$ is the maximum angle of incidence on the lenslet surface. The angle $\theta _i$ was found by assuming that collimated rays are reaching the lenslet directed vertically downward as depicted in Fig. 1. This is a good approximation when $L_{ext}/R \approx 0.4$ [15], which is the ratio that yields the best approximation to a true elliptical collimating lenslet. Retracing the rays from the antenna to the silicon surface, one finds that total internal reflection occurs for $\theta > \pi /3$, so, to first order, $\theta _i = pi/3$. Plugging in the index of refraction of silicon into Eq. 1, we obtain $p<0.23 \lambda$. Applying this to LF-12, LF-34 and MF-12 wavelengths, the maximum pitch in each band should be approximately 695, 425 and 305 $\mu$m, where LF-X and MF-X correspond to the frequency bands of the LF and MF pixels described in Table 1, each with a shortest wavelength of 3028, 1850 and 1338 $\mu$m.

The machinability constraints assumed in this model are given by the laser spot size, splashing and the geometry aspect ratio. The laser etching equipment at UC Berkeley is a JPT M7 100W source with 1 *ns* pulse duration. It can reach a minimum spot size of 25 $\mu$m  according to the manufacturer specifications. Assuming that the tool will be able to achieve twice that size without complications, all of the etched features in the analyzed geometries had a minimum size of 50 $\mu$m. The splashing of silicon during laser ablation is expected to occur at a minimum of 10 $\mu$m from the edge of a etched feature, as seen in [16]. The splashing can cause re-solidified silicon to enter and/or degrade neighboring features, and so, in this study, all features had a separation of 10 $\mu$m between them. Finally, the aspect ratio *height*/*diameter* of simulated features required an upper limit considering that larger proportions become increasingly difficult to manufacture. In [17], trenches with scales comparable to microwave metamaterials and high aspect ratios of 7 and 13 are achieved with a nanosecond pulse laser. These values were used to estimate that machining aspect ratios above 10 would likely be too challenging for the LiteBIRD lenslets.

The transmission at normal incidence through the metamaterial composed of cylindrical holes was calculated for both single-layer and two-layer arrays. Single-layer results were used to quantify the relationship between the cylinder radius and the effective index of refraction of the metamaterial. The best two-layer metamaterial transmission was found using a gradient descent optimization code^1^ which calculates the desired thickness and refractive indices of multi-layer AR for different frequency bands. Using the results from the single layer simulations, the desired AR thickness and refractive index was translated into the depth and radius of the cylindrical holes. These baseline values were used as initial guesses in a HFSS parametric study, which iterated until a minimum 95% transmission was achieved. To account for the lenslet curvature, the impact of the angle of incidence on the transmission power of the 2-layer cylindrical features was calculated.

The conical holes were modeled as single-layer arrays. The cone’s reducing cross-sectional area as a function of depth has the effect of a continuously increasing index of refraction, which is equivalent to infinitely many dielectric layers. The effect of cone radius and depth on the metamaterial transmission were analyzed in a parametric study and the performance of the geometry was compared to an idealized Klopfenstein taper [18] typically used to match impedances on transmission lines over short distances.

The datasets generated during the current study are available from the corresponding author on reasonable request.

## Results

### Cylindrical Features

**Fig. 3:**
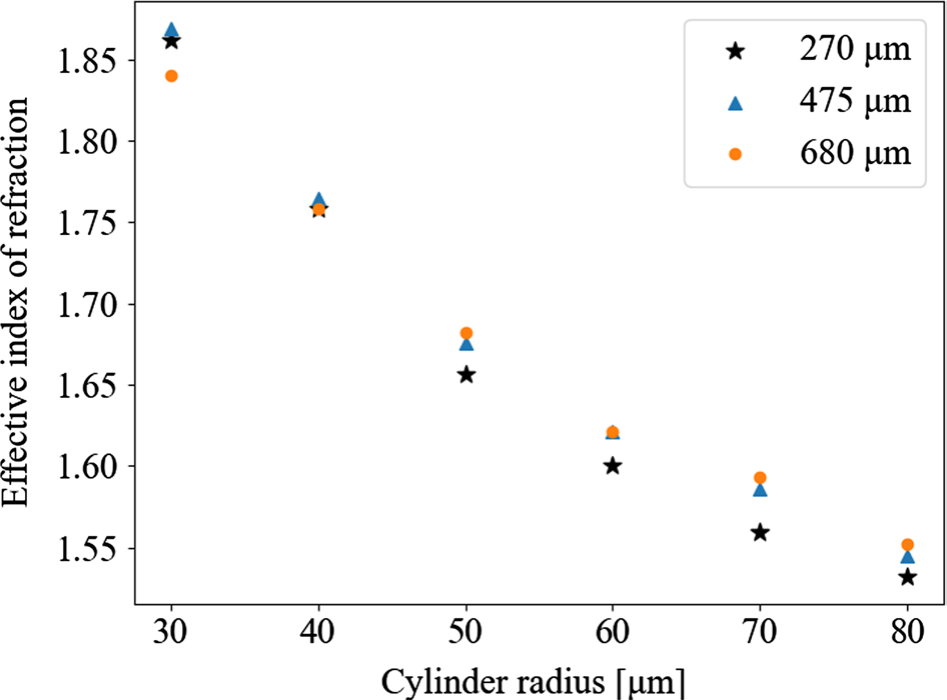
Effective index of refraction of cylindrical features with depths ranging from 270 to 680 $\mu$m. Spacing between features was 10 $\mu$m so that the pitch ranged from 70 to 170 $\mu$m (Color figure online)

The transmission through the single layer of cylindrical holes was calculated for three different hole depths and various radii while maintaining a constant space between features of 10 $\mu$m. The results were compared to the transmission through a single dielectric of same thickness as the cylinder depth, and fitted to find the effective index of refraction ($n_{\rm eff}$) of each cylinder radius. Figure 3 shows that although the effective index of refraction can vary slightly due to changes in depth, the index is mainly a function of the cylinder’s radius.

**Fig. 4:**
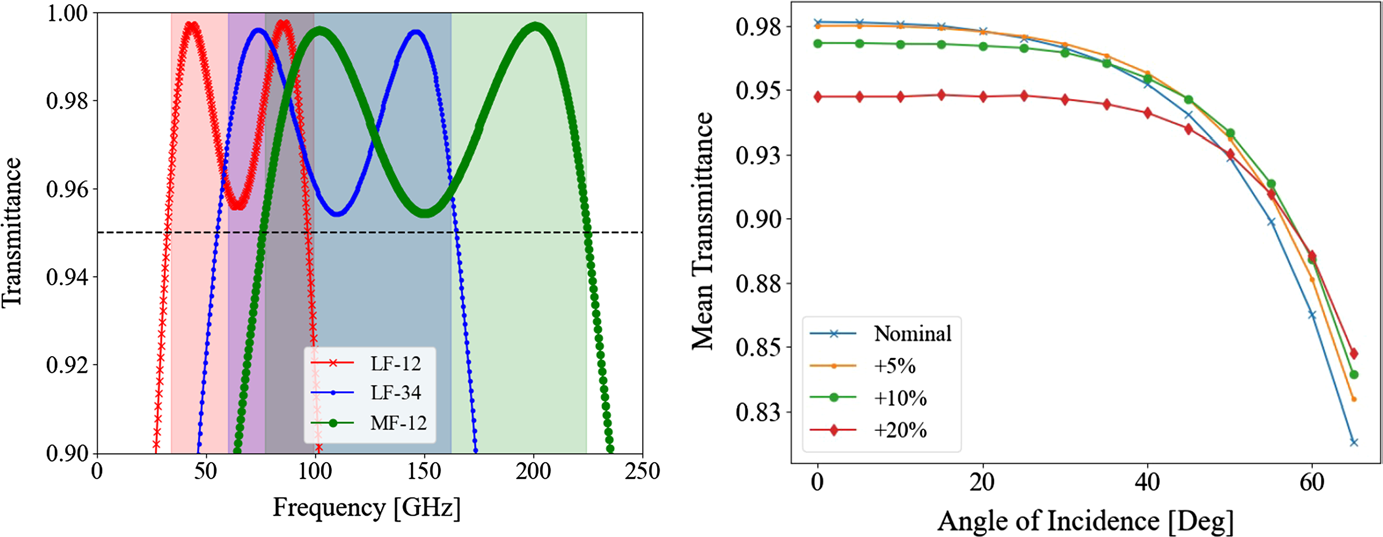
Left: optimized transmission of two-layer cylindrical holes in flat silicon for all LiteBIRD frequency bands using lenslet-coupled antenna arrays. Right: transmission in MF-12 band lenslet across multiple angles of incidence. Each line indicates the transmission achieved by increasing the hole depth by a small factor. Larger hole depth can improve transmission at higher angles of incidence (Color figure online)

The optimized transmission through the two-layer cylindrical metamaterial is shown in Fig. 4a. The transmission in all desired bands was found to be above 95%, with an average of 97.6%, 97.7% and 97.7% in bands LF-12, LF-23 and MF-12, respectively. Table 2 summarizes the geometrical parameters used in the simulation, which can be used as reference for the manufacturing of laser-etched metamaterials in flat silicon, and as baseline for the upcoming analysis in curved surfaces. The tolerance on the feature sizes is of approximately ± 3%. Above that, although the mean transmission can remain higher than 95%, the transmission at the edges of each frequency band begins to change significantly. This translates into desired laser machining tolerances of roughly 5 $\mu$m.

Figure 4b shows the average transmission through the 2-layer cylindrical metamaterial optimized for MF-12 at varying angles of incidence (nominal curve). For angles smaller than 45°, where most of the antenna power is concentrated, the average transmission decreases by a few percent when compared to normal transmission, but remains above 95%. The figure also shows the effect of increasing the metamaterial hole depths by 5, 10 and 20%: namely, deeper features have better transmission at higher angles of incidence, and could potentially be used to optimize transmission across a desired range of angles. Similar trends were found for LF-12 and LF-34 frequencies.

**Table 2:** Recommended geometry for metamaterial AR with two layers of cylindrical features

Name	LF-12	LF-34	MF-12
Frequency band [GHz]	34–99	60–162	77–224
Space between holes (s) [$\mu$m]	10	10	10
Top diameter (TD) [$\mu$m]	160	160	160
Bottom diameter (BD) [$\mu$m]	120	120	120
Top layer height (TH) [$\mu$m]	700	400	290
Bottom layer height (BH) [$\mu$m]	545	325	235
Total height [$\mu$m]	1245	725	525
Pitch (p) [$\mu$m]	170	170	170

### Conical Features

**Fig. 5:**
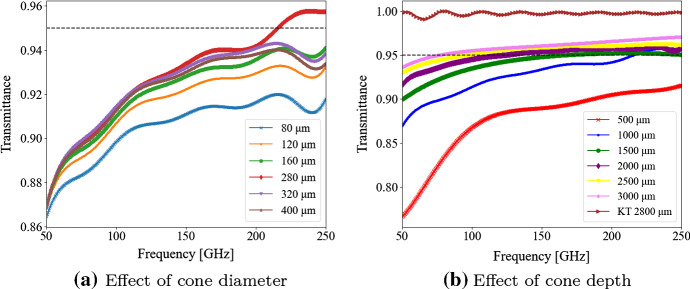
Transmission through a conical metamaterial geometry at normal incidence. Achieving 95% transmission across the 77–224 GHz band requires conical features with high aspect ratios that are likely too challenging to manufacture. Nominal parameters: diameter = 280 $\mu$m, height = 1000 $\mu$m, *s* = 10 $\mu$m. In (**b**), KT refers to the transmission through a theoretical Klopfenstein taper of length 2800 $\mu m$ (Color figure online)

Figure 5 shows the effect of increasing the conical hole diameter (*a*) and depth (*b*) in the 50-250 GHz range. The left panel in Fig. 5 shows that increasing the cone diameter can improve transmission up to a point. However, it is not possible to increase the hole size indefinitely, since, as described in Sect. 2, photons begin to “see” metamaterial features if their size is comparable to the photon wavelength. For MF-12 (77–224 GHz band), the maximum allowed diameter is around 300 $\mu$m, and Fig. 5*b* shows that when we approach that diameter size, the cone depth must be around 3 mm deep to achieve a transmission above 95% across the entire frequency band. At that depth, the aspect ratio (*height*/*pitch*) of the geometry is approaching 10, which can become challenging to manufacture, specially on a curved surface. A comparison to an ideal Klopfenstein taper of length $l = 2800 \, \mu m$, which slightly smaller than the cone’s maximum allowable depth, is included in *b*. The graph shows that the conical shape does not approach the theoretical cap for such a large bandwidth transmission. The taper length was selected by maximizing transmission in MF-12 while keeping $l<3$ mm.

## Conclusion

In this proceeding, we explored the feasibility of manufacturing metamaterial anti-reflection layers in silicon lenslets for the LiteBIRD telescope. Laser etching has the advantage of flexibility in achieving effective dielectric constants through different shapes of structures and of avoiding delamination of dielectrics at cryogenic temperatures due to differential thermal contraction. HFSS simulations on flat silicon showed that two-layer cylindrical holes could be used to achieve transmission above 95% for a wide range of angles of incidence and that single-layer conical holes that achieve this minimum transmittance would likely be too challenging to manufacture. Future work should further explore cylindrical features in non-flat surfaces to further investigate how the lenslet curvature will affect the metamaterial design.

## Footnotes

1 https://github.com/NoahSailer/MetaMat.
